# Deep Convolutional Neural Network Based Interictal-Preictal Electroencephalography Prediction: Application to Focal Cortical Dysplasia Type-II

**DOI:** 10.3389/fneur.2020.594679

**Published:** 2020-11-05

**Authors:** Yoon Gi Chung, Yonghoon Jeon, Sun Ah Choi, Anna Cho, Hunmin Kim, Hee Hwang, Ki Joong Kim

**Affiliations:** ^1^Healthcare ICT Research Center, Seoul National University Bundang Hospital, Seongnam, South Korea; ^2^Department of Pediatrics, Ewha Womans University Medical Center, Ewha Womans University College of Medicine, Seoul, South Korea; ^3^Department of Pediatrics, Seoul National University Bundang Hospital, Seoul National University College of Medicine, Seongnam, South Korea; ^4^Department of Pediatrics, Seoul National University Children's Hospital, Seoul National University College of Medicine, Seoul, South Korea

**Keywords:** epilepsy surgery, seizure prediction, focal cortical dysplasia, deep learning, convolutional neural networks

## Abstract

We aimed to differentiate between the interictal and preictal states in epilepsy patients with focal cortical dysplasia (FCD) type-II using deep learning-based classifiers based on intracranial electroencephalography (EEG). We also investigated the practical conditions for high interictal-preictal discriminability in terms of spatiotemporal EEG characteristics and data size efficiency. Intracranial EEG recordings of nine epilepsy patients with FCD type-II (four female, five male; mean age: 10.7 years) were analyzed. Seizure onset and channel ranking were annotated by two epileptologists. We performed three consecutive interictal-preictal classification steps by varying the preictal length, number of electrodes, and sampling frequency with convolutional neural networks (CNN) using 30 s time-frequency data matrices. Classification performances were evaluated based on accuracy, F1 score, precision, and recall with respect to the above-mentioned three parameters. We found that (1) a 5 min preictal length provided the best classification performance, showing a remarkable enhancement of >13% on average compared to that with the 120 min preictal length; (2) four electrodes provided considerably high classification performance with a decrease of only approximately 1% on average compared to that with all channels; and (3) there was minimal performance change when quadrupling the sampling frequency from 128 Hz. Patient-specific performance variations were noticeable with respect to the preictal length, and three patients showed above-average performance enhancements of >28%. However, performance enhancements were low with respect to both the number of electrodes and sampling frequencies, and some patients showed at most 1–2% performance change. CNN-based classifiers from intracranial EEG recordings using a small number of electrodes and efficient sampling frequency are feasible for predicting the interictal-preictal state transition preceding seizures in epilepsy patients with FCD type-II. Preictal lengths affect the predictability in a patient-specific manner; therefore, pre-examinations for optimal preictal length will be helpful in seizure prediction.

## Introduction

Accurate prediction of an impending seizure can alleviate life-threatening risks in patients when combined with precautions such as alarms, as is common in seizure advisory systems (1). Despite the highly erratic characteristics of an epileptic seizure, numerous studies have reported seizure prediction approaches in the scalp and intracranial electroencephalography (EEG) based on linear and non-linear analysis methods, demonstrating the feasibility of recognizing epileptic seizures in advance (1–4). In addition to the conventional threshold-based and machine-learning-based predictors, recent deep learning-based studies have demonstrated strong seizure prediction by adopting convolutional neural networks (CNN) (5–7), long short-term memory networks (8), mixed models (9, 10), and semi-supervised (11) models with various multivariate spatiotemporal time domain, frequency domain, and time-frequency domain features.

Successful anticipation of a seizure is greatly dependent on an understanding of the neurophysiological transition process from interictal (between seizures) to preictal (prior to seizures) states (2–4). Many studies have investigated the interictal-preictal transition with noticeable changes in linear and non-linear measures of EEG dynamics (12–17). Determining when the transition starts provides meaningful clues to an impending seizure within a certain timeframe, namely the preictal period—the period between the starting point of the transition and seizure onset—which can span from several minutes to hours (2). Recent studies have attempted to determine an optimal length of the preictal period based on probability-statistical approaches in human EEGs (5, 18) and training datasets with varying preictal lengths using machine learning techniques in human (19) and canine EEGs (20).

Previous studies utilizing spatiotemporal analysis approaches have also investigated the preictal dynamic changes in terms of regional effects on seizure prediction (12, 15, 17, 21). Significantly high predictability has been revealed in a seizure onset zone and its surrounding areas through discernible interictal and preictal states of human EEG (22). Nevertheless, these findings remain controversial due to contradictory studies showing predictability beyond the seizure onset areas (23–25). In this context, recent studies have investigated the most preferred EEG recording sites based on combinations of electrodes (25–27) using an entropy-based algorithm (9).

Focal cortical dysplasia (FCD) is the most common etiology in children with intractable epilepsy requiring resective epilepsy surgery. FCDs are classified into type-I, -II, and -III based on histopathological features (28). Patients with FCDs undergo presurgical evaluations using electrophysiology and functional imaging to identify the extent of their epileptogenic zones. Those who suffer from medically refractory seizures are, in some cases, even encouraged to use implantable devices for seizure detection (29, 30) and prediction (31, 32). These implanted detection systems contain enough computational power to allow for long-term EEG monitoring while still assuring the patient's comfort, thereby allowing for timely medical interventions in a patient-specific manner. Several simple, low-power machine learning algorithms have been proposed based on feature extraction from EEG spectral powers (33, 34). Recently, deep learning techniques have been unequivocally identified as superior for capturing the spatiotemporal neurophysiological signatures of preictal states (35).

In this study, we adopted a well-known deep learning approach to discriminate between the interictal and preictal states that precede intractable seizures in epilepsy patients with FCD type-II. We varied (1) the length of preictal periods, (2) the number of EEG electrodes, and (3) sampling frequencies of input data to investigate the practical conditions for high interictal-preictal discriminability in deep learning-based classification models. To our knowledge, this is the first study to demonstrate the feasibility of deep learning-based classifiers to predict the interictal-preictal state transition preceding seizures in epilepsy patients with FCD type-II.

## Materials and Methods

### Dataset

This study was approved by the Institutional Review Board (IRB) of Seoul National University Hospital (IRB No. H-2007-091-1141). The IRB waived the requirement for obtaining informed consent due to the retrospective nature of the review of medical records and EEG data. Patients were identified via electronic medical record review. We retrospectively reviewed all patients who underwent resective epilepsy surgery at Seoul National University Children's Hospital between July 2014 and September 2018. We selected magnetic resonance imaging (MRI) lesional cases with postoperative histological diagnosis of FCD type-II that had intracranial EEG monitoring as a part of the respective surgery. Patients over the age of 19 were excluded. Nine patients (four female, five male; mean age ± standard deviation: 10.7 ± 4.3 years) were included in our analysis. Patients' demographics and clinical characteristics, including electrode number and types, MRI lesion locations, and intracranial ictal EEG patterns (36), were collected. Presurgical intracranial EEG recordings from the nine patients were analyzed. All the recordings were obtained using a digital EEG system (Grass Telefactor Inc., West Warwick, Rhode Island, United States) with the number of subdural and depth electrodes varying from 24 to 78 by patient at a 1,600 Hz sampling frequency. Details of patient clinical information are shown in Table 1.

**Table 1:** Patient clinical information.

**Patient No.**	**Age (years)**	**Sex**	**Electrodes**		**Lesion location**	**Intracranial ictal EEG patterns (36)**
			**Total number**	**Type and number**		
1	12.4	M	24	16 (subdural) 4 × 2 (depth)	Right frontal	Low-voltage fast activities
2	6.6	F	48	40 (subdural) 4 × 2 (depth)	Right temporal	Low-voltage fast activities
3	6.7	M	52	48 (subdural) 4 × 1 (depth)	Right parietal	Burst of high amplitude polyspikes
4	7.6	M	32	32 (subdural)	Left frontal	Sharp activities at <13 Hz
5	18.2	M	58	50 (subdural) 4 × 2 (depth)	Left frontal	Burst of high amplitude polyspikes
6	5.8	F	40	32 (subdural) 4 × 2 (depth)	Left temporal	Sharp activities at <13 Hz
7	15.2	F	40	32 (subdural) 4 × 2 (depth)	Left temporal	Low-voltage fast activities
8	8.9	F	76	68 (subdural) 4 × 2 (depth)	Left frontal	Sharp activities at <13 Hz
9	14.6	M	78	74 (subdural) 4 × 1 (depth)	Right frontal	Low-voltage fast activities

*EEG, electroencephalography; M, male; F, female*.

Two epileptologists (HH and KH) annotated EEG seizure onsets and terminations after re-reviewing whole long-term intracranial video-EEG data. In addition, they provided electrode rankings of the patients based on the clinical importance of electrodes in surgical decision. For example, electrodes on seizure onset zones were considered most important, and those on the other zones of interictal significance—such as zones showing interictal spikes or slow waves—were considered less important. A series of consecutive seizures within 1 h was considered as one seizure. The first seizure in the series was defined as a lead seizure. The EEG recordings from at least 3 h before and after onsets of lead seizures were defined as interictal periods, those from 1 to 120 min prior to onsets of lead seizures were defined as preictal periods, and those from 30 min after the last seizure in the series were defined as postictal periods. The lesser length a preictal period had, the closer it was located to the onset of a lead seizure. A 30 min preictal period was considered as default, whereby at least 60 min (including the 30 min preictal and 30 min postictal periods) were found between two lead seizures.

### Preprocessing

The EEG recordings were down-sampled at 128–512 Hz sampling frequencies, and band-pass filtered between 0.5–50 Hz and 0.5–90 Hz for the down-sampling rates of 128 Hz and 256–512 Hz, respectively. The band-pass filtered signals were segmented into 30 s epochs varying the amount of overlap based on the preictal lengths to equalize the number of preictal and interictal epochs as close as possible. Each epoch was transformed to a time-frequency two-dimensional data matrix by the short-time Fourier transform using a 1 s Hamming window with 50% overlap to capture non-stationary EEG characteristics both in time and frequency domains as seen in recent human (6, 35) and canine (37) studies on the deep learning-based seizure prediction. Coefficients between 55 and 64 Hz were excluded from the time-frequency data matrix down-sampled at 256–512 Hz to remove power line noise effects. In this way, the time-frequency data matrices down-sampled at 128 Hz and 256–512 Hz had dimensions (*n* × T × F) of *n* × 59 × 65 and *n* × 59 × 81, respectively, where n was the number of electrodes.

### Interictal-Preictal Classification

**Figure 1:**
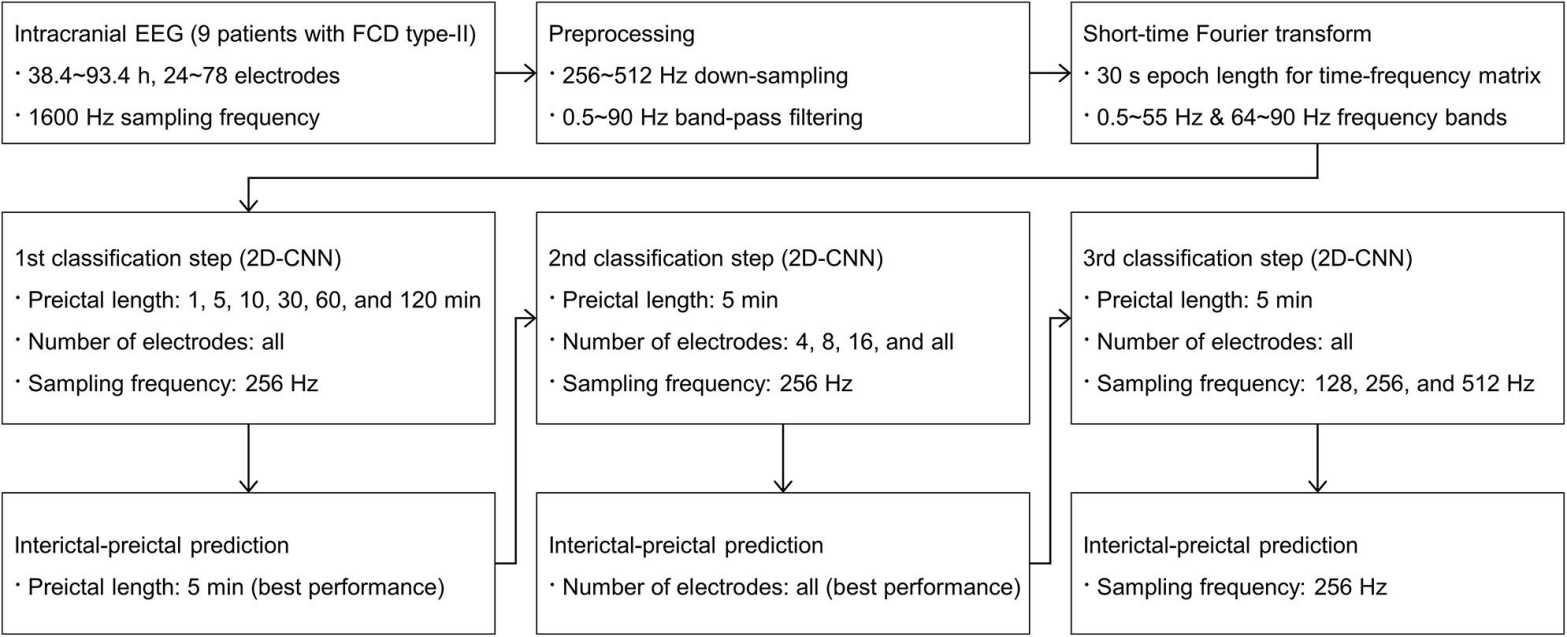
Overall procedures of our proposed consecutive classification steps.

We performed three consecutive interictal-preictal classification steps. Detailed procedures are shown in Figure 1. To deal with an imbalanced distribution of interictal data sets that outnumbered the preictal data sets, a random under-sampling method was applied to make a 1:1 ratio of interictal to preictal data sets. To ensure the robustness and generality of our classification models, all the data for each patient were randomly split into three subsets of training, validation, and test with a ratio of 6:2:2. The total number of samples used for training, validation, and test is shown in Supplementary Table 4.

In the first classification step, preictal lengths were varied from 1, 5, 10, 30, 60, to 120 min for input data to investigate variations in classification performance with respect to the preictal lengths. The EEG recordings from all electrodes, varying from 24 to 78 electrodes implanted for each patient, at a 256 Hz down-sampling frequency were used. For the preictal lengths from 1 to 30 min, the 30 min preictal period set by default was applied to generate input data. For the preictal lengths from 60 to 120 min, 60 and 120 min preictal periods were newly applied, respectively, to generate input data. In each case, lead seizures within 1.5 or 2.5 h including both preictal and postictal periods were considered as one lead seizure. Hence, the number of lead seizures decreased and their corresponding ictal durations increased for the preictal lengths from 30 to 120 min. Likewise, their corresponding interictal durations decreased for those preictal lengths. There was no change in the number of lead seizures and their corresponding ictal and interictal durations in two patients (No. 4 and 9) because all the seizures in those patients were separated sufficiently by more than 2.5 h between seizures. Details of our dataset information, including the number of seizures and lead seizures as well as their corresponding ictal and interictal durations, are shown in Table 2.

**Table 2:** Dataset information.

**Patient No.**	**Total recording time (h)**	**Ictal (h)**	**Inter-ictal (h)**	**Number of seizures**	**Based on preictal lengths and their corresponding lead seizures**								
					**≤30 min**			**60 min**			**120 min**		
					**Ictal (h)**	**Inter-ictal (h)**	**Lead seizures**	**Ictal (h)**	**Inter-ictal (h)**	**Lead seizures**	**Ictal (h)**	**Inter-ictal (h)**	**Lead seizures**
1	63.4	0.99	62.4	84	18.2	45.2	19	21.7	41.8	13	22.2	41.2	5
2	38.4	0.05	38.4	4	1.5	36.9	3	2.4	36.0	2	2.4	36.0	2
3	39.9	0.52	39.4	34	13.8	26.2	13	15.5	24.4	9	18.4	21.6	3
4	93.4	0.03	93.4	8	4.0	89.4	8	4.0	89.4	8	4.0	89.4	8
5	42.3	0.17	42.1	99	10.5	31.8	5	1.6	40.7	2	13.6	28.7	3
6	71.8	0.12	71.7	4	2.1	69.7	4	2.1	69.7	4	1.6	70.2	3
7	59.0	0.38	58.6	38	15.5	43.5	19	24.7	34.2	14	33.7	25.3	7
8	52.0	0.76	51.2	73	20.0	32.1	17	23.3	28.7	11	29.1	22.9	6
9	66.1	0.06	66.0	5	2.6	63.5	5	2.6	63.5	5	2.6	63.5	5
Average	58.5	0.34	58.1	38.8	9.8	48.7	10.3	10.9	47.6	7.6	14.2	44.3	4.7
Total	526.4	3.08	523.3	349	88.1	438.2	93	97.9	428.4	68	127.4	398.9	42

In the second classification step, the number of electrodes was varied from 4, 8, and 16, to all electrodes for input data to investigate variations in classification performance with respect to the number of electrodes. The predetermined preictal length showing the best performance in the first classification step and a 256 Hz down-sampling frequency were applied. Four, eight, and 16 electrodes were selected according to their clinical significance ranking in each patient. Since recent seizure detection (29) and prediction (32) devices have used multiple four-electrode strips, the minimum number of electrodes was set to four.

In the third classification step, down-sampling frequencies were varied from 128, 256, to 512 Hz for input data to investigate variations in classification performance with respect to sampling frequencies and their corresponding data sizes. The EEG recordings from all electrodes were down-sampled at those sampling frequencies separately with the same predetermined preictal length as in the second classification step.

### CNN Architecture

We adopted CNN structures (38) with three convolution blocks using the time-frequency data matrix as an input. Each convolution block consisted of a convolution layer with a batch normalization (39), a rectified linear unit (ReLU) activation function, and a max pooling layer. Batch normalization was used to improve training speed and to reduce overfitting. The first convolution block had 64, *n* × 5 × 5 kernels, where n is the number of electrodes, with a stride of 2. The next two convolution blocks both had 64, 3 × 3 kernels with a stride of 1. Each convolution layer used a 2 × 2 max pooling layer. Following the three convolution blocks, two consecutive fully-connected (FC) layers were used. The former one had a ReLU activation function, while the latter one had a sigmoid activation function. The output size of the latter one and its activation function were 256 and 1, respectively. Dropout (40) was applied to the first FC layer with a probability of 0.5.

All experiments were implemented in Python 3.6 with PyTorch library 1.4.0 (41), using four NVIDIA TITAN V graphics cards with 12 GB memory graphic processing units and CUDA 10.1 programming interface. He initialization (42) was applied to network weights. To train the networks, the RMSProp optimizer was employed with a learning rate, momentum, and weight decay of 5 × 10^−4^, 0.9, and 1 × 10^−6^, respectively. The binary cross-entropy loss function was used as a cost function. Early stopping was used not only to avoid overfitting on the validation set but also to improve our model's generalization ability. As our classification performance was sufficiently high using the validation set, hyperparameters including the number of epochs, learning rate, and patience in early stopping were fixed and no further tuning was required during our validation step. Model performances on the test set were evaluated based on four different measures including accuracy, F1 score, precision, and recall.

## Results

### Preictal Lengths

**Figure 2:**
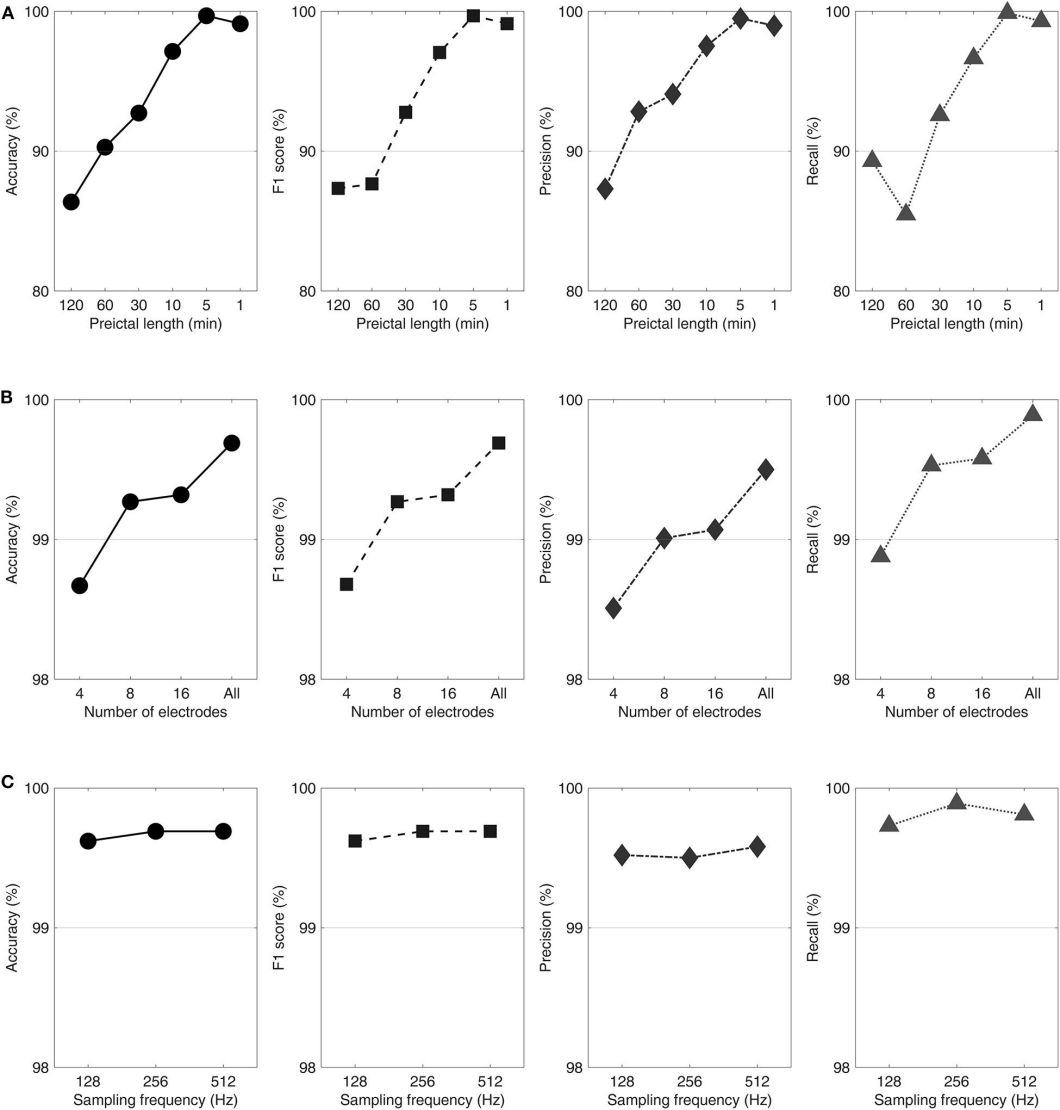
Performance variations with respect to the preictal lengths **(A)**, number of electrodes **(B)**, and sampling frequencies **(C)** shown in accuracy, F1 score, precision, and recall from left to right. Classification procedures were performed varying the preictal lengths with all electrodes and a 256 Hz sampling frequency **(A)**, varying the number of electrodes with a 5 min preictal length and a 256 Hz sampling frequency **(B)**, and varying the sampling frequencies with a 5 min preictal length and using all electrodes **(C)**.

On average, interictal-preictal classification performance was the best at the 5 min preictal length with all electrodes and the 256 Hz sampling frequency in the first classification step. The overall accuracy of classification gradually increased from 86.37 to 99.69%, showing a noticeable performance enhancement of 13.32%, by reducing the preictal length from 120 to 5 min. The F1 score, precision, and recall averaged across patients also showed distinct performance enhancements of 11.78, 11.67, and 9.99%, respectively, by reducing the preictal length from 120 to 5 min. The performance variations with respect to the preictal lengths for each evaluation measure are shown in Figure 2A.

For each preictal length, four (44%), eight (89%), and two patients (22%) showed the highest performance at 1, 5, and 10 min, respectively. Among the eight patients who showed the highest performance at the 5 min preictal length, three patients (No. 1, 4, and 9) showed highly remarkable above-average performance enhancements (28.65–37.73%) by reducing the preictal length from 120 to 5 min. Three patients (No. 3, 5, and 8) were least affected by the decrease in the preictal length from 120 to 5 min, showing a slight increase in performance (<2%).

### Number of Electrodes

On average, classification performance was best with all electrodes using the predefined 5 min preictal length and the 256 Hz sampling frequency in the second classification step. The accuracy averaged over all patients increased from 98.67 to 99.69%, showing a slight performance enhancement of 1.02%, by increasing the number of electrodes from four to all. The patients' average F1 score, precision, and recall showed tiny performance enhancements of 1.01, 0.99, and 1.01%, respectively, when the number of electrodes was increased from four to 24–78 depending on patient. The performance variations with respect to the number of electrodes for each evaluation measure are shown in Figure 2B.

Eight patients (89%) showed the highest performance with all electrodes and less than five patients (<50%) showed the highest performance with 4, 8, and 16 electrodes using the 5 min preictal length. Three patients (No. 3, 7, and 8) maintained the highest performance for all conditions (except for the condition of eight electrodes in No. 7), showing no performance enhancement by increasing the number of electrodes using the 5 min preictal length. Only one patient (No. 4) showed a performance enhancement larger than 2%.

The prediction models required fewer parameters with 60.9, 63.1, and 67.4% of all-channel cases for 4-, 8-, and 16-channel cases, respectively, for one (No. 8) of the three patients who maintained the highest performance for all conditions. The lower the number of electrodes, the higher the model efficiency.

### Sampling Frequencies

On average, classification performance was the best at the 512 Hz sampling frequency using the 5 min preictal length and all electrodes in the third classification step. The patients' average accuracy increased from 99.62 to 99.69%, showing a slight performance enhancement of 0.07%, by increasing the sampling frequency from 128 to 512 Hz. The average F1 score, precision, and recall of patients also showed tiny performance enhancements of 0.07, 0.06, and 0.08%, respectively, after quadrupling the sampling frequency from 128 Hz. The performance variations with respect to the sampling frequencies for each evaluation measure are shown in Figure 2C.

For each sampling frequency, four patients (44%), seven patients (78%), and six patients (67%) showed their highest performances at 128, 256, and 512 Hz, respectively, using the 5 min preictal length and all electrodes. Two patients (No. 3 and 7) maintained their highest performances across all conditions showing no performance enhancement by increasing the sampling frequencies. Five patients showed performance enhancements of <1% (0.15–0.58%). Two patients (No. 4 and 7) showed performance declines of <1% (0.48–0.92%).

The prediction model using 128 Hz required fewer parameters with 86.2% of the cases using 256–512 Hz for one (No. 7) of the two patients who maintained the highest performance for all conditions. The lower the sampling frequency, the higher the model efficiency.

Details of performance variations with respect to the preictal lengths, number of electrodes, and sampling frequencies for each patient are shown in Table 3 and Supplementary Figure 1 (by accuracy). Those for other measures are shown in Supplementary Tables 1–3 for F1 score, precision, and recall, respectively. The number of model parameters in terms of the number of electrodes and sampling frequency are shown in Supplementary Tables 5, 6, respectively.

**Table 3:** Performance variations with respect to the preictal lengths, number of electrodes, and sampling frequencies shown in terms of accuracy (unit: %).

**Patient No.**	**All electrodes, 256 Hz sampling frequency**						**5 min preictal length, 256 Hz sampling frequency**				**5 min preictal length, all electrodes**		
	**Preictal length (min)**						**Electrodes**				**Sampling frequency (Hz)**		
	**120**	**60**	**30**	**10**	**5**	**1**	**4**	**8**	**16**	**All**	**128**	**256**	**512**
1	62.08	83.17	80.22	83.18	99.81	99.13	99.61	99.81	99.71	99.81	99.61	99.81	99.81
2	95.83	99.16	98.87	100.00	100.00	100.00	98.46	98.46	99.38	100.00	99.85	100.00	100.00
3	98.61	98.84	98.87	100.00	100.00	100.00	100.00	100.00	100.00	100.00	100.00	100.00	100.00
4	67.81	56.53	74.37	97.60	98.62	95.62	95.85	98.04	97.58	98.62	99.42	98.62	98.50
5	98.27	98.08	95.21	97.90	99.08	99.59	97.42	99.45	98.34	99.08	99.26	99.08	99.82
6	96.17	94.13	98.63	99.34	99.88	99.74	98.38	99.77	99.77	99.88	99.19	99.88	99.77
7	89.14	96.88	98.90	97.25	100.00	98.80	100.00	99.52	100.00	100.00	100.00	100.00	99.52
8	98.26	97.92	95.31	99.87	100.00	100.00	100.00	100.00	100.00	100.00	100.00	100.00	100.00
9	71.17	87.87	94.23	99.13	99.82	99.17	98.34	98.34	99.08	99.82	99.26	99.82	99.82
Average	86.37	90.29	92.73	97.14	99.69	99.12	98.67	99.27	99.32	99.69	99.62	99.69	99.69

## Discussion

We investigated the practical conditions for high interictal-preictal discriminability in CNN-based classification models using intracranial EEG recordings of epilepsy patients with FCD type-II. We chose the CNN architecture to figure out image-shape EEG signatures in both time and frequency domains to learn short-term time-frequency relationships by convolutional filters and to classify EEG states in an end-to-end fashion (5). We showed that, in our consecutive classification steps, (1) the 5 min preictal length provided the best classification performance producing a remarkable performance enhancement of >13% on average compared to that with the 120 min preictal length, (2) four electrodes provided considerably high classification performance showing a performance decrease of only around 1% on average compared to that with all electrodes, and (3) there was little performance change after quadrupling the sampling frequency from 128 Hz. Our results suggest that CNN-based classifiers using intracranial EEG recordings from a small number of electrodes with an efficient sampling frequency are feasible for predicting the interictal-preictal state transition preceding seizures in epilepsy patients with FCD type-II. Preictal lengths affect the predictability in a patient-specific manner, and therefore, pre-examinations for an optimal preictal length will be helpful in seizure prediction.

### Preictal Lengths

In spite of a large number of studies investigating EEG dynamics, the exact timing and duration of true interictal-preictal state transition remain unclear (12–17, 21, 43–45). Most recent deep learning-based studies typically define their preictal lengths, such as 10 min (5), 15 min (8, 35), 30 min (10), or 60 min (6, 9), in advance of model training. In particular, Khan et al. (5) tried to determine the most favorable preictal length based on a Kullback-Leibler divergence to reflect scalp EEG dynamics on the true state transition. However, predefined preictal lengths are inevitably prone to deviating from actual preictal lengths. Here, we focused on determining how long the preictal period should be by using deep learning-based classification models for high interictal-preictal discriminability, rather than determining when it occurs based on EEG dynamics. Therefore, we suggest that the 5 min preictal length is the most appropriate choice among the CNN-based classification models in our epilepsy patients with FCD type-II, affording the highest interictal-preictal discriminability. We further suggest that the true state transition mostly occurs around 5 min prior to the patient's seizure. In three patients (No. 1, 4, and 9), the state transition may have occurred near the seizure onset because the discriminability increases by reducing preictal lengths. In the other six patients, the state transition may have occurred long before seizure onset, or the EEG characteristics of the interictal and preictal states may have been extremely subtle, because discriminability was high across all preictal lengths. Importantly, when preictal changes occur and whether they can be accurately detected are of critical importance in seizure prediction as well as for accurate application of rescue measures. For example, in patients with sufficient intervals between preictal changes and clinical seizures, rescue medication can be administered in numerous forms, including sublingually, rectally, or intranasally, to maximize the clinical outcome. Other patients with short intervals, such as 5 min, will be limited to parenteral administration or local instillation. Consequently, detailed identification of variations in interictal-preictal discriminability with respect to the preictal lengths can be highly useful for individualized seizure prediction and thus help guide therapeutic approaches.

### Number of Electrodes

Where EEG electrodes should be located and how many electrodes are required for successful seizure prediction are still unclear. For example, some studies have suggested that electrodes close to seizure onset zones have high interictal-preictal discriminability (17), whereas others have observed that electrodes remote to or even contralateral to seizure onset zones also have high discriminability particularly in synchrony-based studies (16, 25, 46). Importantly, the majority of previous deep learning-based studies focused on either all intact electrodes (5, 6, 35), a fraction of all electrodes chosen by a clinical examination (10), or an entropy-based algorithm (9) that was selected in advance of model training. Furthermore, recent seizure advisory systems typically consist of a 16-electrode implantable device based on multiple 4-electrode strips that collect long-term human intracranial EEG recordings for conventional machine learning (31, 32) and deep learning (35) techniques. Finally, cortical stimulation devices designed to detect seizure occurrences are typically made up of 8 or 16 electrodes with multiple 4-electrode strips (29, 30). For these reasons, we investigated the discriminability under conditions of at least four electrodes of clinical importance (near seizure onset zones) in our epilepsy patients with FCD type-II and all intact electrodes both near and far from the seizure onset zones. As all recordings were gathered from one hemisphere, contralateral electrodes were not considered in our study. Interestingly, we found that the discriminability with four electrodes is similar to that with 8, 16, and all electrodes in our patients. Hence, we suggest that a small number of intracranial EEG electrodes, as few as four, are sufficient for high interictal-preictal discriminability by CNN-based classification models in our patients on the condition that the electrodes are located near seizure onset zones and an optimal preictal length is defined. In fact, we only observed around a 1% decline in discriminability when the number of electrodes was reduced to four. However, implantation of intracranial electrode devices is a highly invasive procedure. Therefore, determining the exact amount of decline in interictal-preictal discriminability based on the number of implanted electrodes will help inform epileptologists in selecting the fewest number of electrodes to implant as well as guide manufacturers in designing future devices.

Reduction of the number of electrodes in this study was based on the clinical significance of intracranial EEG monitoring. Electrodes of higher clinical significance, such as those in ictal onset zones or zones with frequent spike appearance were ranked higher in reducing the number of electrodes. This reduction narrows down the zones to presumptive epileptogenic zones (47), which can be more focal in FCD type-II. This procedure should be carefully assessed when evaluating the optimal number of electrodes for other substrates of intractable focal epilepsy. The main purpose of reducing the number of electrodes was to minimize the invasiveness of the implanted commercial device. However, when considering long-term applications of prediction or forecasting devices, slight changes in this parameter can significantly affect performance, such as increased false alarm rates. These caveats should be accurately assessed and carefully addressed when applying our findings in clinical practice.

### Sampling Frequencies

Few studies have been conducted on seizure prediction approaches in terms of varying sampling frequencies and their corresponding data sizes. Using a low sampling frequency as well as a small number of electrodes is a practical strategy for reducing the size of input data in implantable seizure advisory systems. Higher sampling frequencies provide large input data including high-frequency activities exceeding several hundred Hz. Pearce et al. (48) investigated temporal distributions of high-frequency oscillations (HFOs) including ripples and fast ripples in interictal, preictal, ictal, and postictal periods. However, they reported highly varied distributions of the HFOs among patients and poor performance outcomes in their seizure predictors. Recent studies using implantable devices for seizure detection and prediction acquired intracranial EEG recordings at sampling frequencies lower than 400 Hz in human (29–32, 35) and canine (37) subjects, likely to lower power consumption and increase data processing efficiency. Our results suggest that sampling frequencies from 128 to 512 Hz have no significant impact on interictal-preictal discriminability by CNN-based classification models. Given that previous studies have observed preictal signatures in gamma bands (43, 49), 256 Hz is considered to be more proper than 128 Hz because intracranial EEG recordings sampled at the 256 Hz have frequency information up to its Nyquist frequency of 128 Hz including the gamma bands. However, we found that increasing sampling frequency provides minimal improvement on interictal-preictal discriminability which should be taken into consideration in the planning and designing of future implantable devices for seizure prediction.

### Limitations and Suggestions

There are several limitations in this study. The number of patients was small and the locations of FCD type-II as well as the number of seizures were varied. We also used in-house intracranial EEG data recorded under invasive monitoring, which are not representative of the patients' everyday lives. Finally, the ranking of electrodes was arbitrary even though it was performed with the final results of invasive monitoring and re-reviewing of the data. To solve our limitations, we suggest increasing the number of patients and incorporating long-term surgical outcomes into the ranking and selection of electrodes. When considering the future applications of seizure forecasting in intractable focal epilepsy patients, the invasiveness of intracranial electrodes is a major obstacle. Future studies with long-term EEG monitoring using scalp EEGs can solve the invasiveness of intracranial electrodes.

## Conclusion

Deep learning techniques have been strongly suggested as a novel seizure prediction approach because no hand-engineered feature extraction procedure is required to explore complicated spatiotemporal neurophysiological markers. This study showed that intracranial EEG recordings from a small number of electrodes near seizure onset zones using a sampling frequency sufficient to detect gamma bands can successfully discriminate interictal-preictal states preceding seizures in epilepsy patients with FCD type-II. Importantly, this requires the determination of an optimal preictal length using CNN-based classification models. Our findings can be considered a preliminary proof of concept for the feasibility of deep learning-based seizure prediction systems. For our next steps, we plan to evaluate the predictability of seizure prediction systems based on our deep learning-based classification models using long-term intracranial EEG recordings in a larger number of epilepsy patients with FCD type-II.

## Data Availability Statement

The original contributions presented in the study are included in the article/Supplementary Material, further inquiries can be directed to the corresponding author/s.

## Ethics Statement

The studies involving human participants were reviewed and approved by Institutional Review Board (IRB) of Seoul National University Hospital (IRB No. H-2007-091-1141). Written informed consent from the participants' legal guardian/next of kin was not required to participate in this study in accordance with the national legislation and the institutional requirements.

## Author Contributions

HK, HH, and KK: conceptualization and design of study. HK, SC, and AC: clinical interpretation. YC, YJ, and HK: data acquisition and analysis. YC, YJ, SC, and HK: drafting and revision of the manuscript. All authors contributed to the article and approved the submitted version.

## Conflict of Interest

The authors declare that the research was conducted in the absence of any commercial or financial relationships that could be construed as a potential conflict of interest.
