# Supplementary material for: Deep Convolutional Neural Network Based Interictal-Preictal Electroencephalography Prediction: Application to Focal Cortical Dysplasia Type-II
Source: Front Neurol. 2020 Nov 5;11:594679. doi: 10.3389/fneur.2020.594679 (PMC7674929; doi:10.3389/fneur.2020.594679)
Supplement: Supplementary file 1 [file Data_Sheet_1.docx]

Supplementary Material

# Supplementary Figure


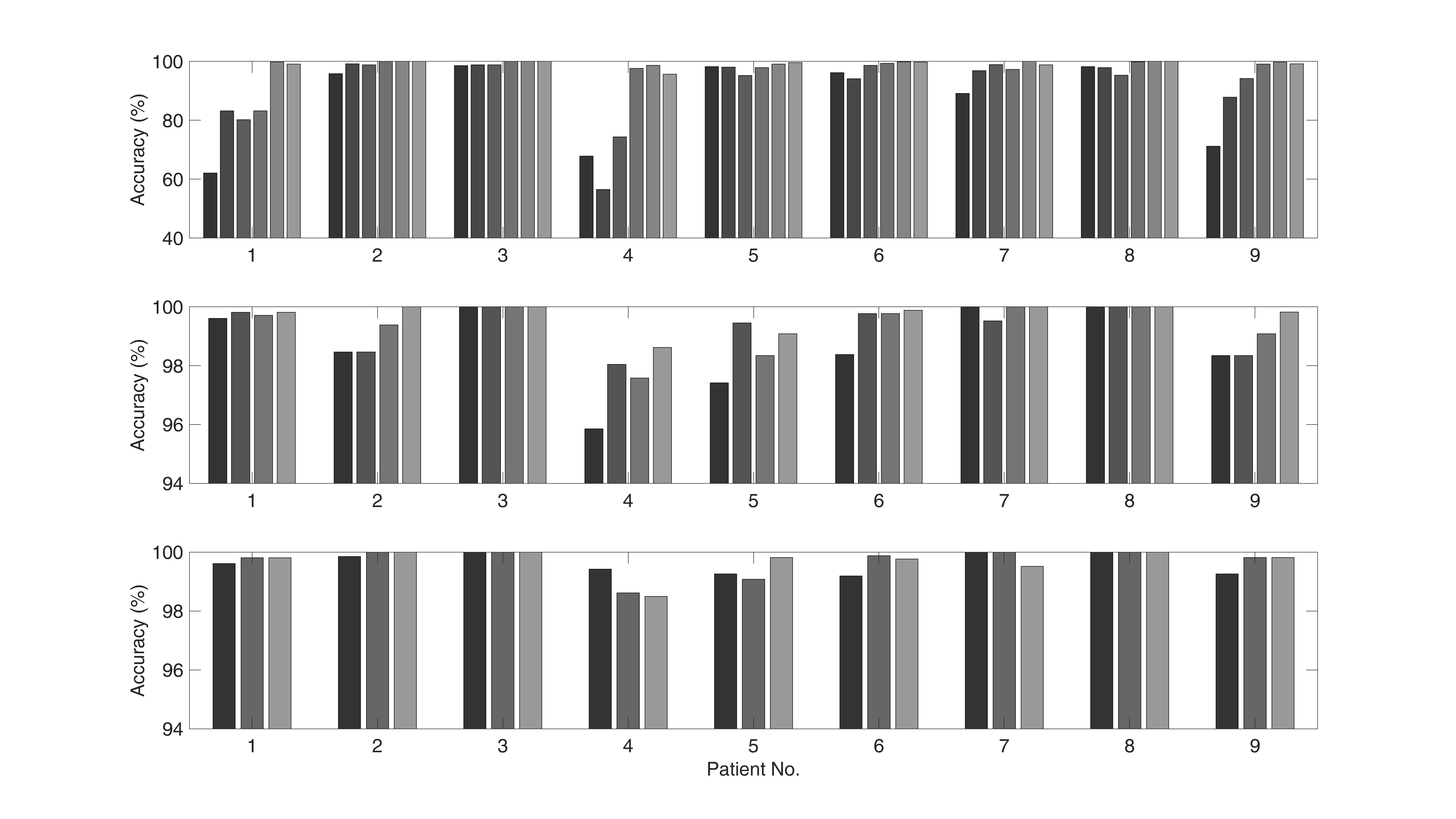


Figure S1. Patient-specific performance variations with respect to the preictal lengths (top), number of electrodes (middle), and sampling frequencies (bottom) shown in terms of accuracy. Bars from left to right represent 120, 60, 30, 10, 5, and 1 min preictal lengths (top); 4, 8, 16, and all electrodes (middle); and 128, 256, and 512 Hz sampling frequencies (bottom).

# 2. Supplementary Tables

Table S1. Performance variations with respect to the preictal lengths, number of electrodes, and sampling frequencies shown in terms of F1 score (unit: %).

| Patient No. | All electrodes, 256 Hz sampling frequency | | | | | | 5 min preictal length, 256 Hz sampling frequency | | | | 5 min preictal length, all electrodes | | |
| --- | --- | --- | --- | --- | --- | --- | --- | --- | --- | --- | --- | --- | --- |
|  | Preictal length (min) | | | | | | Electrodes | | | | Sampling frequency (Hz) | | |
|  | 120 | 60 | 30 | 10 | 5 | 1 | 4 | 8 | 16 | All | 128 | 256 | 512 |
| 1 | 71.91 | 83.17 | 76.72 | 82.48 | 99.81 | 99.12 | 99.61 | 99.81 | 99.71 | 99.81 | 99.61 | 99.81 | 99.81 |
| 2 | 95.71 | 99.16 | 98.87 | 100.00 | 100.00 | 100.00 | 98.44 | 98.47 | 99.39 | 100.00 | 99.85 | 100.00 | 100.00 |
| 3 | 98.61 | 98.85 | 98.87 | 100.00 | 100.00 | 100.00 | 100.00 | 100.00 | 100.00 | 100.00 | 100.00 | 100.00 | 100.00 |
| 4 | 64.43 | 33.58 | 78.55 | 97.63 | 98.63 | 95.74 | 95.93 | 98.05 | 97.60 | 98.63 | 99.42 | 98.63 | 98.51 |
| 5 | 98.29 | 98.08 | 95.09 | 97.90 | 99.08 | 99.59 | 97.39 | 99.45 | 98.36 | 99.08 | 99.26 | 99.08 | 99.82 |
| 6 | 96.18 | 93.89 | 98.64 | 99.34 | 99.88 | 99.74 | 98.41 | 99.77 | 99.77 | 99.88 | 99.19 | 99.88 | 99.77 |
| 7 | 87.81 | 96.77 | 98.90 | 97.18 | 100.00 | 98.79 | 100.00 | 99.52 | 100.00 | 100.00 | 100.00 | 100.00 | 99.52 |
| 8 | 98.23 | 97.94 | 95.10 | 99.87 | 100.00 | 100.00 | 100.00 | 100.00 | 100.00 | 100.00 | 100.00 | 100.00 | 100.00 |
| 9 | 74.92 | 87.54 | 94.28 | 99.13 | 99.82 | 99.17 | 98.37 | 98.35 | 99.08 | 99.82 | 99.27 | 99.82 | 99.82 |
| Average | 87.34 | 87.66 | 92.78 | 97.06 | 99.69 | 99.13 | 98.68 | 99.27 | 99.32 | 99.69 | 99.62 | 99.69 | 99.69 |

Table S2. Performance variations with respect to the preictal lengths, number of electrodes, and sampling frequencies shown in terms of precision (unit: %).

| Patient No. | All electrodes, 256 Hz sampling frequency | | | | | | 5 min preictal length, 256 Hz sampling frequency | | | | 5 min preictal length, all electrodes | | |
| --- | --- | --- | --- | --- | --- | --- | --- | --- | --- | --- | --- | --- | --- |
|  | Preictal length (min) | | | | | | Electrodes | | | | Sampling frequency (Hz) | | |
|  | 120 | 60 | 30 | 10 | 5 | 1 | 4 | 8 | 16 | All | 128 | 256 | 512 |
| 1 | 57.11 | 83.07 | 93.35 | 86.07 | 99.81 | 99.78 | 100.00 | 99.61 | 99.61 | 99.81 | 99.61 | 99.81 | 99.81 |
| 2 | 98.53 | 99.58 | 99.15 | 100.00 | 100.00 | 100.00 | 100.00 | 97.87 | 99.08 | 100.00 | 100.00 | 100.00 | 100.00 |
| 3 | 98.61 | 98.62 | 100.00 | 100.00 | 100.00 | 100.00 | 100.00 | 100.00 | 100.00 | 100.00 | 100.00 | 100.00 | 100.00 |
| 4 | 72.09 | 71.34 | 67.53 | 96.19 | 97.74 | 93.17 | 94.22 | 97.71 | 96.83 | 97.74 | 99.31 | 97.74 | 97.95 |
| 5 | 97.30 | 97.91 | 97.63 | 97.90 | 98.54 | 99.18 | 98.49 | 99.26 | 97.12 | 98.54 | 98.90 | 98.54 | 99.63 |
| 6 | 95.85 | 97.95 | 97.33 | 99.56 | 99.77 | 99.74 | 97.08 | 99.54 | 99.77 | 99.77 | 99.31 | 99.77 | 99.77 |
| 7 | 100.00 | 100.00 | 98.90 | 99.76 | 100.00 | 99.78 | 100.00 | 99.61 | 100.00 | 100.00 | 100.00 | 100.00 | 99.42 |
| 8 | 100.00 | 97.03 | 99.46 | 100.00 | 100.00 | 100.00 | 100.00 | 100.00 | 100.00 | 100.00 | 100.00 | 100.00 | 100.00 |
| 9 | 66.30 | 90.00 | 93.37 | 98.28 | 99.63 | 99.17 | 96.79 | 97.46 | 99.26 | 99.63 | 98.55 | 99.63 | 99.63 |
| Average | 87.31 | 92.83 | 94.08 | 97.53 | 99.50 | 98.98 | 98.51 | 99.01 | 99.07 | 99.50 | 99.52 | 99.50 | 99.58 |

Table S3. Performance variations with respect to the preictal lengths, number of electrodes, and sampling frequencies shown in terms of recall (unit: %).

| Patient No. | All electrodes, 256 Hz sampling frequency | | | | | | 5 min preictal length, 256 Hz sampling frequency | | | | 5 min preictal length, all electrodes | | |
| --- | --- | --- | --- | --- | --- | --- | --- | --- | --- | --- | --- | --- | --- |
|  | Preictal length (min) | | | | | | Electrodes | | | | Sampling frequency (Hz) | | |
|  | 120 | 60 | 30 | 10 | 5 | 1 | 4 | 8 | 16 | All | 128 | 256 | 512 |
| 1 | 97.08 | 83.33 | 65.12 | 79.18 | 99.81 | 98.48 | 99.23 | 100.00 | 99.81 | 99.81 | 99.61 | 99.81 | 99.81 |
| 2 | 93.06 | 98.74 | 98.59 | 100.00 | 100.00 | 100.00 | 96.92 | 99.08 | 99.69 | 100.00 | 99.69 | 100.00 | 100.00 |
| 3 | 98.61 | 99.07 | 97.74 | 100.00 | 100.00 | 100.00 | 100.00 | 100.00 | 100.00 | 100.00 | 100.00 | 100.00 | 100.00 |
| 4 | 58.24 | 21.96 | 93.88 | 99.13 | 99.54 | 98.45 | 97.70 | 98.39 | 98.39 | 99.54 | 99.54 | 99.54 | 99.08 |
| 5 | 99.31 | 98.25 | 92.68 | 97.90 | 99.63 | 100.00 | 96.31 | 99.63 | 99.63 | 99.63 | 99.63 | 99.63 | 100.00 |
| 6 | 96.52 | 90.15 | 100.00 | 99.12 | 100.00 | 99.74 | 99.77 | 100.00 | 99.77 | 100.00 | 99.08 | 100.00 | 99.77 |
| 7 | 78.27 | 93.75 | 98.90 | 94.74 | 100.00 | 97.83 | 100.00 | 99.42 | 100.00 | 100.00 | 100.00 | 100.00 | 99.61 |
| 8 | 96.53 | 98.86 | 91.11 | 99.74 | 100.00 | 100.00 | 100.00 | 100.00 | 100.00 | 100.00 | 100.00 | 100.00 | 100.00 |
| 9 | 86.12 | 85.21 | 95.21 | 100.00 | 100.00 | 99.17 | 100.00 | 99.26 | 98.89 | 100.00 | 100.00 | 100.00 | 100.00 |
| Average | 89.30 | 85.48 | 92.58 | 96.65 | 99.89 | 99.30 | 98.88 | 99.53 | 99.58 | 99.89 | 99.73 | 99.89 | 99.81 |

Table S4. The total number of samples used for training, validation, and test.

| Patient No. | Preictal length (min) | | | | | | | | | | | | | | | | | |
| --- | --- | --- | --- | --- | --- | --- | --- | --- | --- | --- | --- | --- | --- | --- | --- | --- | --- | --- |
|  | 120 | | | 60 | | | 30 | | | 10 | | | 5 | | | 1 | | |
|  | Tr | Val | Te | Tr | Val | Te | Tr | Val | Te | Tr | Val | Te | Tr | Val | Te | Tr | Val | Te |
| 1 | 1440 | 480 | 480 | 1872 | 624 | 624 | 2712 | 905 | 905 | 2622 | 874 | 874 | 3100 | 1034 | 1034 | 2758 | 920 | 920 |
| 2 | 1722 | 575 | 575 | 1430 | 477 | 477 | 2127 | 709 | 710 | 2055 | 685 | 686 | 1947 | 649 | 650 | 1731 | 577 | 578 |
| 3 | 864 | 288 | 288 | 1296 | 432 | 432 | 1856 | 619 | 619 | 1794 | 598 | 598 | 2121 | 707 | 708 | 1887 | 629 | 630 |
| 4 | 3054 | 1019 | 1019 | 3054 | 1019 | 1019 | 2841 | 947 | 948 | 2745 | 915 | 916 | 2601 | 867 | 868 | 1161 | 387 | 388 |
| 5 | 1734 | 579 | 579 | 1716 | 572 | 572 | 2130 | 710 | 710 | 1716 | 572 | 572 | 1626 | 542 | 542 | 1446 | 482 | 482 |
| 6 | 2584 | 862 | 862 | 2860 | 954 | 954 | 2836 | 946 | 946 | 2740 | 914 | 914 | 2596 | 866 | 866 | 2308 | 770 | 770 |
| 7 | 2016 | 672 | 672 | 2016 | 672 | 672 | 2712 | 905 | 905 | 2622 | 874 | 874 | 3100 | 1034 | 1034 | 2758 | 920 | 920 |
| 8 | 1728 | 576 | 576 | 1584 | 528 | 528 | 2427 | 809 | 810 | 2346 | 782 | 782 | 2774 | 925 | 925 | 2468 | 823 | 823 |
| 9 | 2505 | 835 | 836 | 2028 | 676 | 676 | 2130 | 710 | 710 | 1716 | 572 | 572 | 1626 | 542 | 542 | 1446 | 482 | 482 |

* Tr, Val, and Te represent training, validation, and test, respectively.

Table S5. The number of model parameters in terms of the number of electrodes.

| Patient No. | 5 min preictal length, 256 Hz sampling frequency | | | | | | | |
| --- | --- | --- | --- | --- | --- | --- | --- | --- |
|  | Number of parameters | | | | Ratio (%) | | | |
|  | Electrodes | | | | Electrodes | | | |
|  | 4 | 8 | 16 | All | 4 | 8 | 16 | All |
| 1 | 179521 | 185921 | 198721 | 211521 | 84.9 | 87.9 | 93.9 | 100.0 |
| 2 | 179521 | 185921 | 198721 | 249921 | 71.8 | 74.4 | 79.5 | 100.0 |
| 3 | 179521 | 185921 | 198721 | 256321 | 70.0 | 72.5 | 77.5 | 100.0 |
| 4 | 179521 | 185921 | 198721 | 224321 | 80.0 | 82.9 | 88.6 | 100.0 |
| 5 | 179521 | 185921 | 198721 | 265921 | 67.5 | 69.9 | 74.7 | 100.0 |
| 6 | 179521 | 185921 | 198721 | 237121 | 75.7 | 78.4 | 83.8 | 100.0 |
| 7 | 179521 | 185921 | 198721 | 237121 | 75.7 | 78.4 | 83.8 | 100.0 |
| 8 | 179521 | 185921 | 198721 | 294721 | 60.9 | 63.1 | 67.4 | 100.0 |
| 9 | 179521 | 185921 | 198721 | 297921 | 60.3 | 62.4 | 66.7 | 100.0 |

Table S6. The number of model parameters in terms of sampling frequency.

| Patient No. | 5 min preictal length, all electrodes | | | | | |
| --- | --- | --- | --- | --- | --- | --- |
|  | Number of parameters | | | Ratio (%) | | |
|  | Sampling frequency (Hz) | | | Sampling frequency (Hz) | | |
|  | 128 | 256 | 512 | 128 | 256 | 512 |
| 1 | 178753 | 211521 | 211521 | 84.5 | 100.0 | 100.0 |
| 2 | 217153 | 249921 | 249921 | 86.9 | 100.0 | 100.0 |
| 3 | 223553 | 256321 | 256321 | 87.2 | 100.0 | 100.0 |
| 4 | 191553 | 224321 | 224321 | 85.4 | 100.0 | 100.0 |
| 5 | 233153 | 265921 | 265921 | 87.7 | 100.0 | 100.0 |
| 6 | 204353 | 237121 | 237121 | 86.2 | 100.0 | 100.0 |
| 7 | 204353 | 237121 | 237121 | 86.2 | 100.0 | 100.0 |
| 8 | 261953 | 294721 | 294721 | 88.9 | 100.0 | 100.0 |
| 9 | 265153 | 297921 | 297921 | 89.0 | 100.0 | 100.0 |
